# Feedstock Development for Material Extrusion-Based Printing of Ti6Al4V Parts

**DOI:** 10.3390/ma15186442

**Published:** 2022-09-16

**Authors:** Ralf Eickhoff, Steffen Antusch, Siegfried Baumgärtner, Dorit Nötzel, Thomas Hanemann

**Affiliations:** 1 Institute for Applied Materials, Karlsruhe Institute of Technology, Hermann-von-Helmholtz-Platz 1, D-76344 Eggenstein-Leopoldshafen, Germany; 2 Department of Microsystems Engineering, University Freiburg, Georges-Koehler-Allee 102,D-79110 Freiburg, Germany

**Keywords:** material extrusion (MEX), FFF/FFD, 3D printing, additive manufacturing, titanium alloys, Ti6Al4V

## Abstract

In this work, a holistic approach for the fabrication of dense Ti6Al4V parts via material extrusion methods (MEX), such as fused filament fabrication (FFF) or fused feedstock deposition (FFD), will be presented. With respect to the requirements of the printing process, a comprehensive investigation of the feedstock development will be described. This covers mainly the amount ratio variation of the main binder components LDPE (low-density polyethylene), HDPE (high-density polyethylene), and wax, characterized by shear and oscillation rheology. Solid content of 60 vol% allowed the 3D printing of even more complex small parts in a reproducible manner. In some cases, the pellet-based FFD seems to be superior to the established FFF. After sintering, a density of 96.6% of theory could be achieved, an additional hot isostatic pressing delivered density values better than 99% of theory. The requirements (mechanical properties, carbon, and oxygen content) for the usage of medical implants (following ASTM F2885-17) were partially fulfilled or shortly missed.

## 1. Introduction

Within the last 10 years, the different variants of additive manufacturing methods (often denoted as 3D printing) of polymers, metals, and ceramics have revolutionized modern prototyping, tooling, and manufacturing. Beginning almost 40 years ago with polymers and later extending to the other main material classes, many different methods have been commercialized. Some techniques are initially specialized to one material class, such as VAT photopolymerization (stereolithography (SLA), digital light processing, DLP)) for curable resins, material extrusion MEX (fused filament fabrication (FFF)) for polymer melts, powder bed fusion (selective laser sintering (SLS), selective laser melting (SLM), or electron beam melting (EBM)) for metals, respectively. Quite often, some of the listed 3D printing methods are used and partially commercialized, adapting their process characteristics to other materials, such as SLA for ceramics, FFF for ceramics, and metals in analogy to powder injection molding (PIM) [1,2,3,4,5,6,7,8,9,10,11,12].

FFF has several advantages (with respect to applications):

- A simple processing technique from polymer melt processing;
- Almost identical machine setups for polymers and polymer matrix composites;
- Professional and reliable FFF printers;
- Open-source FFF printers suitable for combined material–process development.

Almost all commercially available thermoplastics, even high-performance ones, such as PEEK (polyether ether ketone) a.o., can be printed nowadays in very good quality regarding surface appearance or geometric accuracy. In addition, within the last couple of years, FFF has been extended to the fabrication of ceramic or metal parts modifying the established PIM process chain:

1. The selection of a suitable binder system enabling good flow properties in a wide temperature range, ideally spherical fillers, and surfactants with pronounced impacts on viscosity reduction fulfilling the special needs in FFF as low pressure and a low shear-shaping method.
2. Feedstock compounding with at least 50 vol% (ceramic) or 60 vol% (metal) solid loading.
3. Different extrusion printing methods: filament-based FFF or pellet-based fused feedstock deposition (FFD) and derived techniques.
4. Greenbody postprocessing: debinding and sintering.

With respect to different FFF variants, ceramic parts made from alumina, zirconia, and even a two-component FFF of zirconia with stainless steel were described [13,14,15,16,17,18,19,20,21,22]. In contrast to ceramics, a minor number of references dealing with metal printing via MEX methods [23,24,25,26] are available, a comprehensive overview can be found in [25]. Focusing on process optimization and applications, the fabrication of NdFeB-magnets was described in [27]. Quite recently, studies on the extrusion of stainless steel [28] and low-alloy steel have been published [29]. Lightweight and medical applications are very challenging fields due to the need for outstanding mechanical properties in comparison with low weight, long-time stability, and reliability, as well as biomedical compatibility. One promising material is titanium and the related alloys with the most prominent representative Ti6Al4V. Additive manufacturing should allow for a customized generation of medical implants after solving the AM-specific technical challenges, such as accessible density and the maximum of allowed carbon or oxygen content preserving the aspired mechanical properties [30]. Within the last couple of years, the processing of Ti6Al4V with direct energy methods, such as SLS, SLM, or EBM, has been reported [31,32,33,34]. Extrusion-based AM of titanium and Ti-based alloys is also gaining importance [35,36,37,38,39].

In addition to standard shear stress or shear rate-dependent melt rheology, it is helpful to investigate the viscoelastic behavior of the different feedstock systems by oscillation rheometric measurements. Only a few publications have provided deeper insight into the impact of the complex rheological behavior on the FFF printing technique [20,21,39]. This method allows for the determination of the complex shear modulus G* covering the contributions of the storage modulus G’ and the loss modulus G″ (1). In a more detailed interpretation, G’ is equivalent to the stored energy during the application of a shear force generating any shear stress. In case the strain fades, the stored energy is released and acts as the driving force to move the material back to the pristine position [40]. The loss modulus G″ stands for the energy that is dissipated under shearing conditions. Amplitude sweep measurements allow for the estimation of the yield point, where the viscous flow (G‘‘) starts dominating the elastic behavior (G’) [41]. The knowledge about the viscoelastic flow is relevant for the deposition of a newly printed layer on the previous one and the selection of the printing parameters. Under given printing conditions, the freshly deposited printed material must not remelt the previously printed layer completely, otherwise, the structural integrity is damaged. This may happen if the loss modulus dominates the storage modulus. If the opposite situation is given, this means the existence of a yield point and small deformations originated by the new layer deposition do not affect the surface stability. The presence of a yield point is favorable for FFF and FFD.

G* = G’ + iG″(1)

The aim of this work was to establish the additive manufacturing of Ti6Al4V parts by MEX methods retaining the enhanced quality criteria for use in medical technology. In addition, the applicability of FFD as a suitable alternative to the established FFF will be elaborated.

## 2. Materials and Methods

### 2.1. Material Selection: Feedstock Composition

As in previous work covering the realization of dense ceramic parts [14,15,16,21] via FFF, a process chain originally adapted from PIM [42,43] has been used. Targeting potential applications in medical technology or as healthcare products, such as implants, the established light metal alloy Ti6Al4V (Grade 23FE, Heraeus, Hanau, Germany) was selected [44]. Due to the very particular requirements in medical engineering with enhanced mechanical properties, this grade shows a reduced carbon and oxygen content according to the ASTM F2885-17 standard [30], describing the specifications for metal injection molding (MIM)-produced Ti6Al4V surgical implant applications. The principal binder composition consists of polyethylene (PE and wax), but for a more accurate relation of feedstock composition and final part properties, a low-density polyethylene (LDPE, Lupolen PE1800H, LyondellBasell, Frankfurt, Germany), and high-density polyethylene (HDPE, Hostalen GD4775, LyondellBasell, Frankfurt, Germany) as mayor polymer components were selected. The addition of the low molecular variant of polyethylene, namely paraffin wax (Sasolwax 6403, Sasol GmbH, Hamburg, Germany), ensures a low melt viscosity at moderate temperatures during compounding and printing. In contrast to the previously investigated ceramics [14,15,16,21], the specific surface area (SSA) of the used metal filler is significantly smaller by a factor of 50–90. Therefore, the addition of the different surfactants is not referenced to the SSA; but a constant value of 5 wt% of the whole binder amount is taken as surfactant and subtracted from the low molecular weight paraffin wax amount. Three different surfactants were investigated:

(a) Stearic acid (SA, Carl Roth GmbH, Karlsruhe, Germany) as used previously;
(b) PAT-77/P (E. u. P. Wuertz GmbH & Co. KG, Bingen, Germany), recommended by the vendor for MIM;
(c) PAT-659/CB (E. u. P. Wuertz GmbH & Co. KG, Bingen, Germany), recommended by the vendor for MIM.

SA is an established surfactant in MIM and FFF, as described, e.g., in [38], applying different feedstock mixtures. If no other surfactant is denoted in the text explicitly, 5 wt% SA is used as the standard surfactant in all cases.

All relevant particle properties were measured by

- Particle size distribution by laser diffraction (LA-950 Horiba Ltd., Kyoto, Japan);
- SSA via the BET method (Gemini VII 2390, Micromeritics Instr. Corp., Norcross, GA, USA);
- Particle density via Helium pycnometry (Pycnomatic ATC, Porotec, Germany);
- Particle morphology: SEM Supra 55 (Zeiss, Oberkochen, Germany).

### 2.2. Compounding

All feedstocks were prepared in a mixer-kneader compounder (W50-EHT, Brabender GmbH, Duisburg, Germany) with simultaneous torque recording during mixing. The blade’s rotating speed was set to 30 rpm, and the processing time to 1 h. The compounding temperature depended on the binder composition and was set to the following values: LDPE/wax: 125 °C, HDPE/wax, and HDPE/LDPW/wax: 160 °C. The sequence of material added to the mixing chamber (volume 45 mL) was identical for all feedstock compositions guaranteeing identical processing conditions.

### 2.3. Flow Behavior

The feedstocks were characterized by two complementary methods. The temperature and shear rate dependent melt flow were measured by a high-pressure capillary rheometer (Rheograph 25, Goettfert Werkstoff-Pruefmaschinen GmbH, Buchen, Germany). The following measuring parameters were used:

- Measuring temperature: 160 °C;
- Capillary length and diameter: 30 and 1 mm;
- Shear rate range: 1 to 5000 1/s.

The viscoelastic flow for the determination of the yield point was detected by oscillatory measurements in the amplitude sweep (AS) mode by applying a rotary rheometer (Gemini HR Nano, Netzsch-Gerätebau GmbH & Co. Holding KG, Selb, Germany) equipped with plate–plate geometry (20 mm diameter, gap 1 mm, smooth surface). The following parameters were applied:

- Measuring temperature: 160, 180, 200 °C;
- Shear stress: 0.01–50,000 (Pa);
- Frequency: 1 Hz;
- Pre-shearing: 0.2 1/s.

### 2.4. Extrusion-Based Shaping

Due to the different rheological behaviors of the investigated feedstocks, the following feedstock and shaping method combinations were employed:

- FFF: LDPE/wax; HDPE/LDPE/wax;
- FFD: HDPE/wax, HDPE/LDPE/wax;
- Injection molding: HDPE/wax.

#### 2.4.1. Fused Filament Fabrication (FFF)

An impact mill (Granulator 1514, Rapid Germany, Kleinostheim, Germany) was used to pelletize the obtained feedstock after compounding. The pellets were transformed into filaments by using a single screw filament extruder (Noztek pro HT, Noztek, Shoreham, UK) applying different feedstock composition-related extrusion parameters. A commercial FFF printer (×350pro, German RepRap, Feldkirchen, Germany) was used for printing, applying different extruder nozzles (0.3 and 0.4 mm). To obtain better print results, some machine modifications were done:

- Printhead: increase of the filament diameter from 1.75 to 2.85 mm;
- Installation of additional part surface ventilation;
- Covering the platform with PE-coating spring steel for better part adhesion while printing and part removal after printing;
- Usage of the Ultimaker slicer software Ultimaker Cura (V 4.10.0).

#### 2.4.2. Fused Feedstock Deposition (FFD)

As a new variant of FFF, the processing of pellets as in MIM was possible in FFD. The two-component FFD-printer FFD 150H (3d-figo GmbH, Salzkotten, Germany) possesses two small extruders placed above the printheads; the printer was used in the single component-printing mode. The nozzle diameter was set to a fixed value of 0.4 mm, the platform was covered with PE-coating spring steel. The feedstock pellets were preheated in the extruder to accelerate the extrusion process. In addition to Ultimaker Cura, the slicer Repetier-Host (V 2.2.2) was applied.

#### 2.4.3. Metal Injection Molding

For comparison, similar specimens (cuboids and discs) were fabricated by MIM employing the micro-injection molding machine MS50 (Wittmann-Battenfeld, Koettingbrunn, Austria) using identical feedstocks, such as in FFF and FFD.

### 2.5. Thermal Postprocessing

The debinding process was subdivided into two steps. First, a solvent debinding (50 °C, 24 h) in n-hexane allowed for the removal of the low molecular weight paraffin wax and stearic acid. The liquid pre-debinding was performed in a homemade glass reactor extraction apparatus equipped with a reflux condenser. Second, thermal debinding was conducted prior to sintering in a metal sinter furnace (MUT Advanced Heating GmbH, Jena, Germany). The maximum debinding temperature was 550 °C and the sintering temperature was set to 1350 °C (in both cases in an argon atmosphere, avoiding any oxygen contamination). With respect to enhanced density values, hot isostatic pressing (HIP2000, Dieffenbacher GmbH, Eppingen, Germany) of the selected samples was applied subsequently after sintering.

### 2.6. Characterization

**Figure 1:**
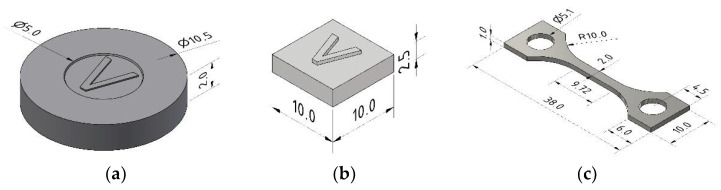
Geometric dimensions (mm) of all specimens: (**a**) disc; (**b**) cuboid; (**c**) tensile testing. The “V” indicates the printing direction. The cuboid and the disc were also injection-molded (without a “V” structure).

All specimen density measurements were conducted by applying a precision balance (Secura 225D-1S) equipped with an Archimedes density set (YDK 01, both Sartorius Lab Instruments GmbH & Co KG, Göttingen, Germany). The specimen’s geometric dimensions were measured by a caliper (VRZ1, Dr. J. Heidenhain GmbH, Traunreut, Germany). The determination of the carbon and oxygen content was carried out by combustion analysis (CS600 and TC600, LECO Instruments GmbH, Mönchengladbach, Germany). The metallographic preparation was done with ATM Brilliant 200 (ATM Qness GmbH, Mammelzen, Germany) using a corundum circular saw followed by resin embedding (Opal 410, ATM Qness GmbH, Mammelzen, Germany). After solidification, the specimens were ground with SiC sandpaper (granularity 320 and 800). Finally, the surface was polished with diamond paste (9 µm) and finished with a mixture of Eposil M (colloidal SiO_2_, 0.06 µm) and a 10% H_2_O_2_ solution on an ATM SAPHIR 350 polishing machine (ATM Qness GmbH, Mammelzen, Germany). Some samples were additionally etched with ammonium hydrogen fluoride. The mechanical tests were investigated under ambient conditions using a tensile testing machine (Z100, Zwick Roell GmbH, & Co KG, ULM, Germany) equipped with the extensometer PMA13/V7/1 (Maytec Mess- und Regeltechnik GmbH, Singen, Germany). The testing speed was set to 0.015 mm/s. The dimensions of all specimens can be seen in Figure 1.

## 3. Results and Discussion

### 3.1. Material Characterization

**Figure 2:**
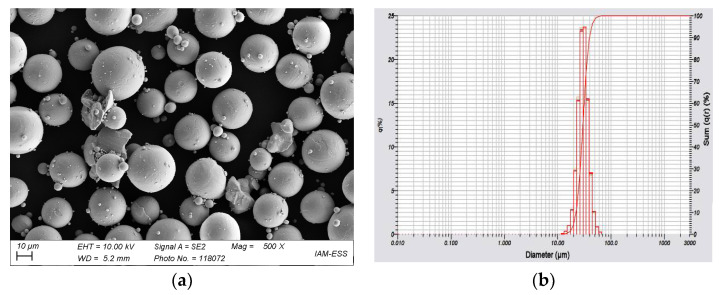
(**a**) SEM-Image of used Ti6Al4V; (**b**) measured particle size distribution.

With respect to the requirements of FFF, FFD, and injection molding, solid loading of Ti6Al4V in a feedstock of at least 60 vol% has to be achieved. A highly-filled feedstock is possible if the particle morphology is spherical, enabling enhanced packing. Figure 2a shows a SEM image of the particle’s morphology. The spherical particle shape is typical for gas atomized metals [45]. Figure 2b contains the measured particle size distribution. A monomodal shape in the range between 10 and 70 µm can be seen. A closer look at the material properties shows an average particle size of around 30 µm (Table 1). The measured values are in good agreement with the vendor data. In contrast to ceramics, the SSA with 0.15 m²/g is quite small.

### 3.2. Compounding

#### 3.2.1. LDPE/wax Binder System

**Figure 3:**
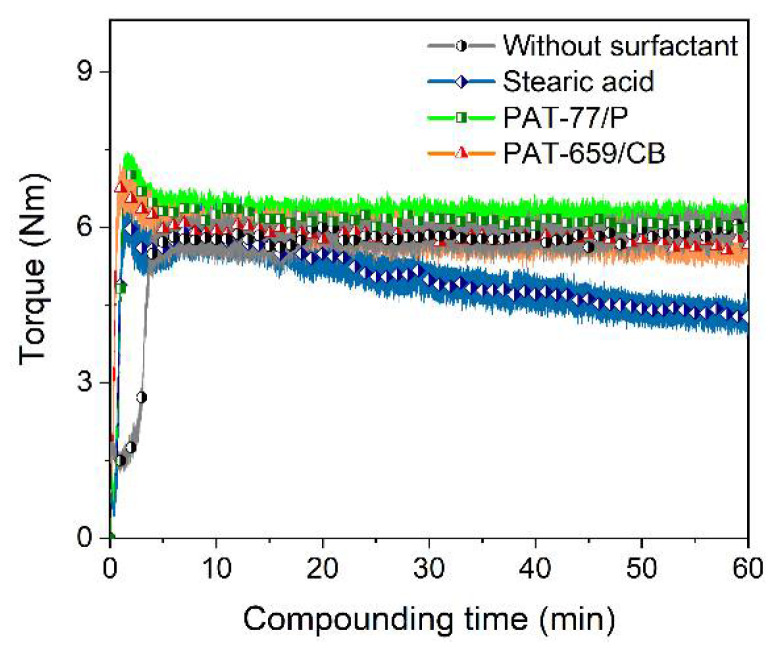
Time-dependent torque during compounding at 125 °C of LDPE/wax-based binder with different surfactants (5 wt%) and a solid load of 60 vol%.

As in previous work on the FFF-printing of alumina containing feedstocks [15] or micro-injection molding of 17-4PH [43], a binder composition of LDPE and wax (ratio 1 to 1) in combination with SA as the surfactant was applied. As stated in the experimental section, the surfactant partially replaced the wax moiety due to similar flow properties. Two commercially available surfactants with unknown compositions designed for MIM were considered. Figure 3 presents the recorded compounding torque of the LDPE/wax/surfactant mixtures. The compounding curve can be split into three phases [46]:

1. Filling phase (addition of all components into the mixing chamber);
2. Mixing phase (agglomerate disruption and particle wetting);
3. Equilibrium phase (the stationary state with simultaneous wetting and dewetting).

Due to the relatively low solid load, the maximum torque of the filling phase is small, and after a short torque decay, the stationary stage is reached delivering a small continuous torque value. Within the experimental error, no significant difference between systems without any surfactant and the two surfactants from E. u. P. Wuertz can be seen. Only the addition of stearic acid caused some small torque drop with time. The limited impact of the surfactants can be attributed to the small solid load and the spherical particle morphology. With respect to FFF of Ti6Al4V feedstocks, other groups used planetary mixers for compounding, applying PLA or a more complex binder system consisting of poly(ethylene-vinyl acetate (EVA)), poly(propylene-ethylene), and polyisobutene with SA as a surfactant [38]. The maximum solid load was around 60–65 vol%. Singh and coworkers investigated a Ti6Al4V containing feedstock with a proprietary binder, prepared with a solid load of 59 vol% in a mixer–kneader system [36,37]. Bek et al. applied a mixer–kneader system as well for feedstock consisting of 60 vol% Ti6Al4V, dispersed in a polypropylene–ethylene copolymer blended with a maleic acid anhydride functionalized polypropylene as the main binder components [39]. In the case of feedstock development for MIM, a partially water-soluble binder made from PEG/PMMA with pure titanium as filler was also reported [47]. An attempt to correlate the mixing method, rheological properties, and filament extrudability for gas atomized Ni-Cu in a PLA matrix was conducted by Hasib et al. [48].

#### 3.2.2. HDPE/Wax Binder System

**Figure 4:**
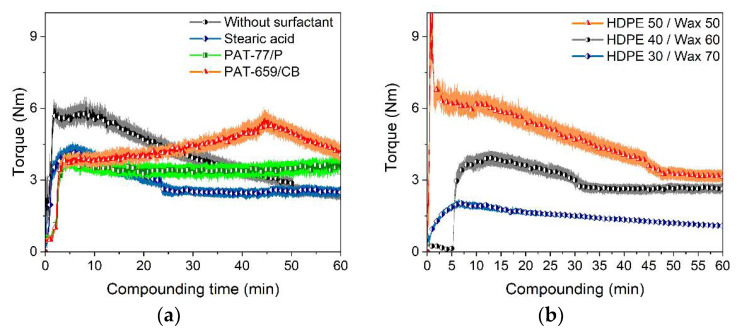
Comparison of different binder systems containing 60 vol% Ti6Al4V. (**a**) Time-dependent torque during compounding at 160 °C of HDPE/wax-based binder with different surfactants; (**b**) compounding of HDPE/wax at 160 °C with the binder component ratio variation (5 wt% SA as surfactant).

The substitution of LDPE with HDPE should cause two major binder property changes. In the solid state, the mechanical strength is higher, supporting demolding (injection molding) and the stability of the previously printed layer in FFF or FFD. Another positive side effect is the enhanced stability of the HDPE-containing binder during solvent pre-debinding. As a drawback, the binder viscosity can be higher at a constant temperature affecting the molding or printing temperature. This can be compensated by changing the HDPE/wax ratio towards increasing wax amounts. Figure 4 compares the impact of different surfactants and HDPE/wax ratios on the compounding behavior and resulting torque with the proceeding compounding time. Comparing the HDPE-based composites with the related LDPE composites, the torque progression is more nonuniform (Figure 4a). Whilst the torque for the compounds without any surfactant or with SA drops with time, the addition of the two PATs causes a torque increase. As a result, these two surfactants were omitted for FFF/FFD printing trials.

The variation of the HDPE/wax ratio has a strong impact on the resulting compounding behavior (Figure 4b). A higher wax amount allows very fast stationary behavior at very low torque values. The substitution of LDPE with HDPE forced a pronounced elevation of the compounding temperature from 125 °C to 160 °C for comparable processing conditions. Despite the fact that SA has a melting temperature of around 70 °C and decomposes at around 360–370 °C, considerable mass losses at temperatures > 160 °C were reported [38]. Therefore, further feedstock processing should not exceed 160 °C.

In general, HDPE is seldomly used in FFF. A recent publication described the printing of a composite prepared by mechanical mixing of HDPE filled with up to 75 wt% cardboard dust [49].

#### 3.2.3. HDPE/LDPE/Wax Binder Systems

**Figure 5:**
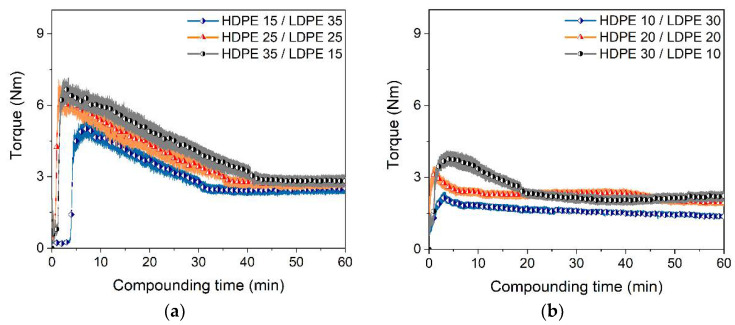
Time-dependent torque measurements during compounding at 160 °C and a solid load of 60 vol%. (**a**) Wax content 50%; (**b**) wax content 60% (5 wt% SA as surfactant).

Another possibility for the adjustment of the compounding behavior towards simple processing and low torque values is the combination of LDPE with HDPE and the variation of the PE/wax ratio. Figure 5a shows the influence of the HDPE/LDPE ratio at a wax content of 50% on the resulting torque values at 160 °C. With increasing HDPE content, the torque values were elevated as well. Surprisingly, it took between 30 and 40 min until the stationary phase was reached, where the final torque differences were almost negligible. A further increase of the wax content to 60% accelerated the compounding process; again, the differences between the various LDPE/HDPE compositions were quite small (Figure 5b).

#### 3.2.4. Solid Load Variation

**Figure 6:**
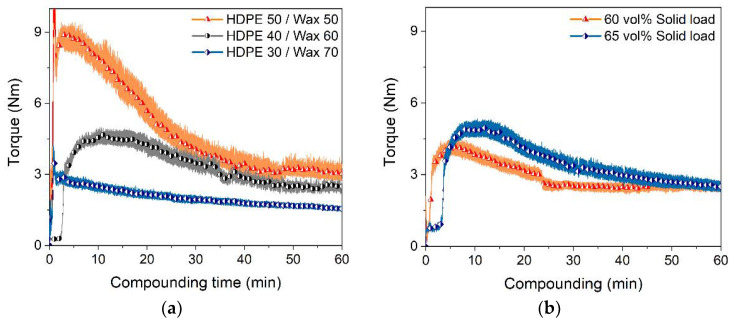
Time-dependent torque measurements during compounding at 160 °C; (**a**) solid load 65 vol% and different HDPE/wax ratios; (**b**) comparison of 60% and 65 vol% solid loads at an HDPE/wax ratio of 40/60.

With respect to dense metal parts, the solid load in the feedstock should be as high as possible. According to the previous results, feedstocks with 65 vol% Ti6Al4V loads were prepared. As expected, the compounding torque increased. Figure 6a shows the change of the torque with time and different HDPE/wax ratios. Increasing wax content lowered the maximum torque value at the end of the filling phase from 9 to 3 Nm with a low final torque value for the stationary phase. For better comparison, Figure 6b shows—at a binder composition of HDPE/wax of 40/60—the compounding curves for 60 vol% and 65 vol%. In the latter case, the compounding phases 1 (mixing) and 2 (wetting) were prolonged, but the final stationary torque values after 1 h were almost identical. As a first resume, the variations of the individual amounts of LDPE, HDPE, and wax enabled tailoring of the compounding behavior; even higher solid loadings were possible.

### 3.3. Flow Behavior

#### 3.3.1. LDPE/Wax Binder System

**Figure 7:**
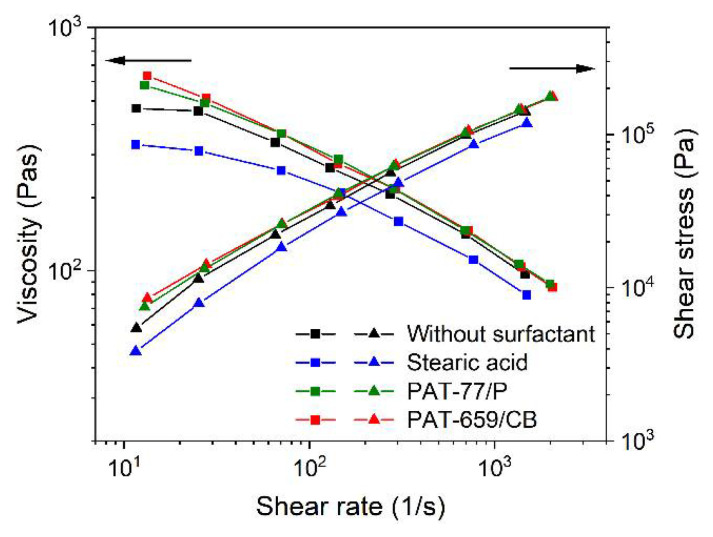
Shear rate-dependent viscosity at 160 °C of LDPE/wax-based binder with different surfactants (5 wt%) and a solid load of 60 vol%.

Knowledge about the rheological behavior as a function of the composition is essential for the first assessment of a feedstock that could be used in FFF, FFD, or injection molding. Figure 7 shows for the LDPE/wax-based binder and different surfactants, the shear-rate dependent viscosity at 160 °C. A similar behavior, such as in the related time-dependent torque change during compounding can be observed (Figure 3). In general, all systems possess a pronounced pseudoplastic flow. The addition of the two commercial additives, PAT-77/P and PAT-659/CB, caused a slight viscosity increase at lower shear rates relative to the reference mixture without any surfactant. Only the presence of SA allowed a pronounced viscosity drop over the whole investigated shear rate range. Interestingly, the viscosity values for a feedstock containing 50 vol% zirconia in the same binder were higher by a factor of almost 10 at small shear rates and the same measuring temperature [21]. This can be attributed to the ceramic´s smaller particle size (d_50_ = 1 µm) and the larger SSA (6.6 m²/g). The pronounced pseudoplastic flow favors injection molding with the related large injection speeds and pressures accompanied by huge shear rates around 1000 1/s or higher and hampers 3D printing by FFF or FFD being a low-pressure shaping method with small shear rates (~100 1/s).

**Figure 8:**
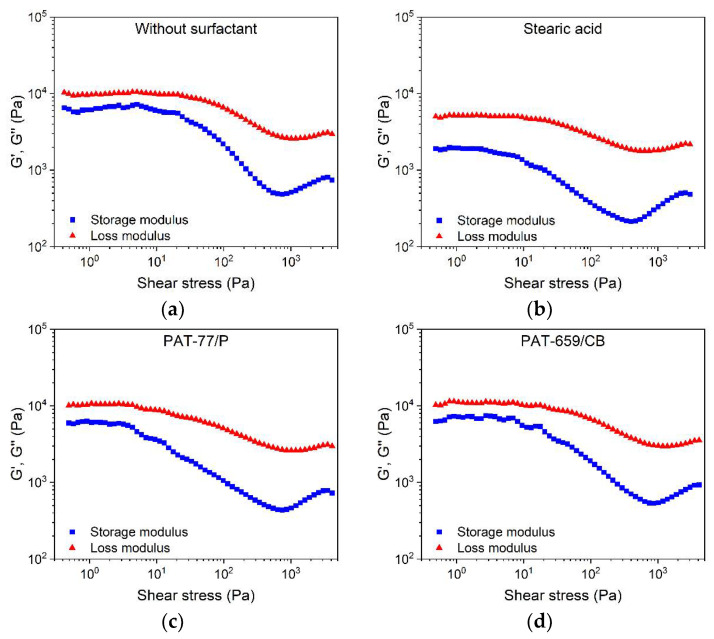
Storage modulus G′ and loss modulus G″ of the LDPE/wax systems containing different surfactants, measured at 160 °C (solid load 60 vol%) via the oscillatory test applying the amplitude sweep; (**a**) without any surfactant; (**b**) stearic acid; (**c**) PAT-77/P; (**d**) PAT-659/CB.

A feedstock containing solid and liquid components represents a heterogeneous material system, including materials of non-unique polarity as well as the solid and liquid state of matter in the melt. Therefore, more complex rheology beyond the ideal flow can be expected. A closer insight into the viscoelastic flow behavior can be obtained by oscillation rheology showing the storage G′ and the loss modulus G″ separately, representing, on the one hand, the more solid-state elastic behavior (G′), and on the other hand, the more liquid flow behavior (G″) (Figure 8). In all four investigated cases, the loss modulus was larger than the storage modules, which gives strong evidence for the dominance of the more viscous part of the complex modulus. Relative to the reference feedstock without surfactant (Figure 8a), only the addition of SA delivered a remarkable drop in both moduli (Figure 8b). In contrast to the vendor’s recommendation, the addition of the two commercial surfactant mixtures PAT-77/P (Figure 8c) and PAT-659/CB (Figure 8d) caused a pronounced increase in the storage modulus G′. This underlines the results obtained in the high-pressure capillary viscosity measurements shown in Figure 7 and may be attributed to a pronounced attractive interaction in the feedstock system.

All investigated feedstocks show (with increasing shear stress) a drop of G′ and G″ followed by a slight increase. One possible explanation is the occurrence of phase separation between the binder and solid filler accompanied by stick-slip behavior. Gloeckle et al., for their different complex feedstock systems (starting almost at a low solid loading of 30 vol%), described a change from the elastic to a more viscous behavior at the applied measuring temperature of 120 °C [38]. Bek et al., for their feedstock, found a change from elastic to viscous flow at a solid load of 60 vol% [39]. A collection of different empirical models usable for the prediction of the flow behavior of MIM feedstocks as functions of the solid load can be found in [50]. As a consequence of the negative impact on the feedstock´s flow behavior and further usage of the two PAT surfactants for FFF/FFD, printing experiments were omitted. One has to consider that the impact of a surfactant depends on the polarity of the filler and the polymer matrix. If a more polar binder is selected, different behavior could be observed.

#### 3.3.2. HDPE/Wax Binder System

**Figure 9:**
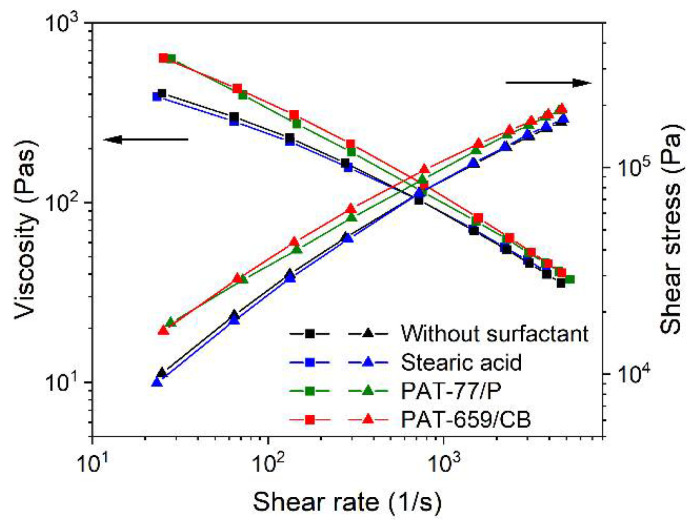
Shear rate-dependent viscosity at 160 °C of the HDPE/wax-based (40/60 ratio) binder with different surfactants (5 wt%) and a solid load of 60 vol%.

The substitution of the branched LDPE with the non-branched HDPE causes an improvement in the mechanical properties, which should enable enhanced green body stability after printing. The more rigid molecular structure leads to an increase in the melting temperature from 108 °C (LDPE) to 130 °C (HDPE), which agrees with data from the literature [51]. Considering the results from compounding (Figure 4b), a HDPE to wax ratio of 40 to 60 was selected for further investigation, which is a compromise between good compounding behavior and green body stability. Because of the HDPE usage instead of LDPE, the viscosity measurements must be performed at higher temperatures to achieve comparable viscosities as in the LDPE-based feedstocks (Figure 7 and Figure 9).

**Figure 10:**
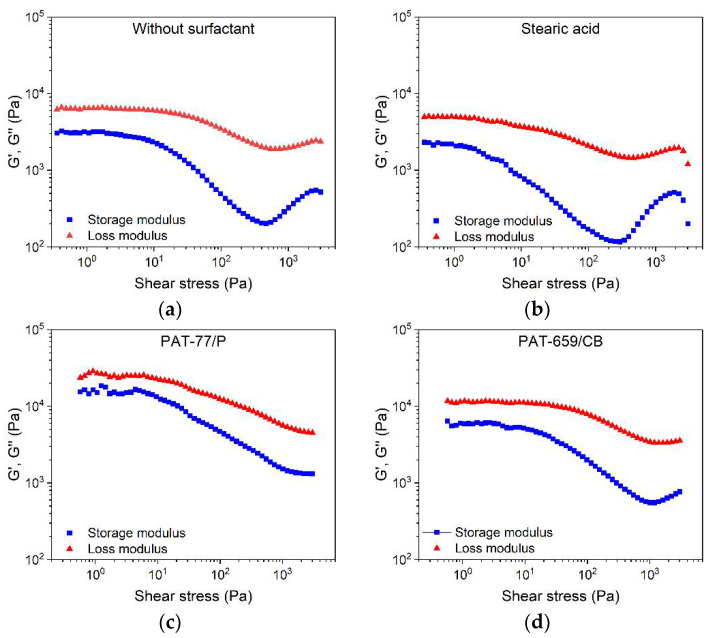
Storage modulus G′ and loss modulus G″ of HDPE/wax systems containing different surfactants, measured at 160 °C (solid load 60 vol%) via the oscillatory test applying the amplitude sweep (40/60 ratio); (**a**) without any surfactant; (**b**) stearic acid; (**c**) PAT-77/P; (**d**) PAT-659/CB.

**Figure 11:**
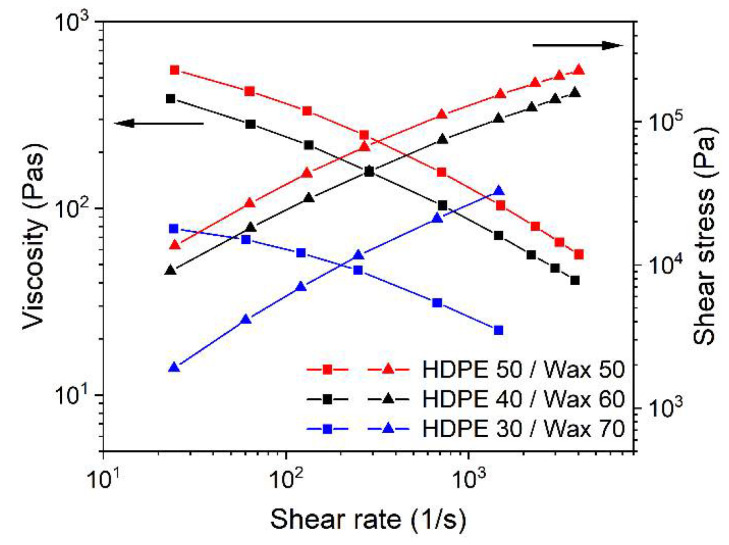
Shear rate-dependent viscosity of HDPE/wax at 160 °C with the binder component ratio variation (5 wt% SA as surfactant).

The surfactant screening delivered similar results as in the LDPE-based mixtures. All feedstocks showed a pronounced pseudoplastic flow and only the addition of SA allowed a viscosity drop, but the positive effect was quite small. In contrast, the viscosity increase by the usage of the two PAT surfactants was stronger than in the LDPE-based mixtures relative to the reference mixtures. The investigation of the surfactant influence on the viscoelastic flow delivered similar results (Figure 10). In all cases, the loss modulus dominated, and a small positive effect of SA and the pronounced negative effect of the two PATs on the storage modulus, such as in the LDPE-based feedstocks, could be seen. The shape of the stress-dependent moduli curve gives evidence for stick-slip behavior and possible phase separation at higher stress values. The feedstock systems did not show any yield point, which means a crossing of the storage with loss moduli. The absence of any yield point can cause difficulties during printing when a fresh layer is deposited on a previously printed one, causing surface destruction by smearing due to a dominating viscous flow effect. As in the LDPE-based feedstocks, the two PAT surfactants were not considered further for FFF/FFD printing experiments. The variation of the HDPE/wax ratio had a pronounced impact on the melt viscosity (Figure 11). A reduction of the HDPE amount from 50% to 30% caused a viscosity drop by (almost) a factor of 10 at lower and moderate shear rates. As a drawback, increasing wax amounts may result in a pronounced reduction of the green body stability and the stability of previously printed layers during the deposition of a new layer.

#### 3.3.3. HDPE/LDPE/Wax Binder System

**Figure 12:**
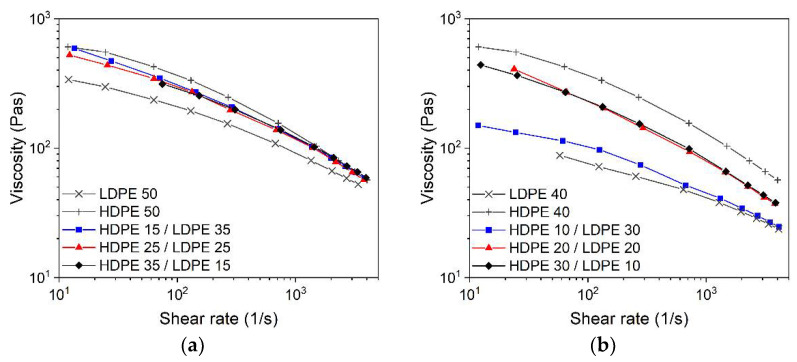
Shear rate-dependent viscosity at 160 °C and a solid load of 60 vol% and HDPE/LDPE ratio variation (5 wt% stearic acid as surfactant); (**a**) wax content 50%; (**b**) wax content 60%.

Finally, a further flow behavior adjustment can be reached by the combination of LDPE with HDPE and with wax as the low molecular weight component. Figure 12a shows (at a 50% wax content in the binder) the viscosity as a function of the HDPE/LDPE ratio and the pure HDPE and LDPE with 50% wax as references. The impact of the ratio is quite small. At a higher wax amount of 60%, a pronounced change in the viscosity with the HDPE/LDPE ratio can be observed (Figure 12b). Zhao et al. investigated LDPE/HDPE/wax feedstocks with yttria-stabilized zirconia as a solid filler (load 85.5 wt%) designed for injection molding [51]. The wax content was set to a fixed value and the ratio of LDPE and HDPE varied. Beyond SA, more additives were included; the compounding occurred in a twin-screw extruder at temperatures around 180–190 °C. In agreement with the results presented here, the substitution of LDPE with HDPE caused only a slight melt viscosity increase, retaining the pronounced pseudoplastic flow [51].

#### 3.3.4. Solid Load Variation

**Figure 13:**
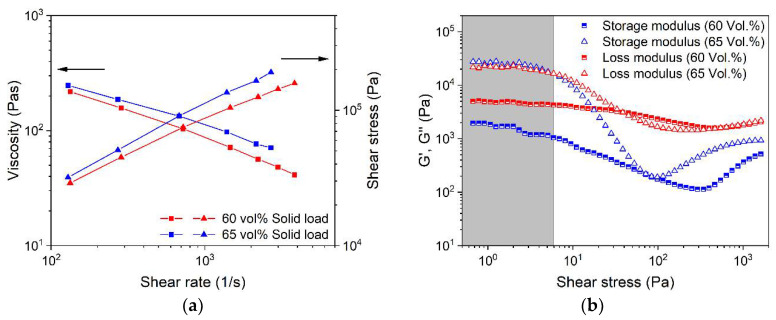
Rheological investigations on a binder system with **the** HDPE/wax ratio 40/60 at 160 °C (5 wt% SA); (**a**) shear rate-dependent viscosity at different solid loads; (**b**) storage modulus G′ and loss modulus G′′ at different solid loads via oscillatory tests applying an amplitude sweep.

With respect to dense parts after sintering, the solid load should be as high as possible; however, quite often, higher loadings cause significant negative impacts on the printing behaviors in FFF. Figure 13a shows the shear rate dependent flow behavior at solid loadings of 60 vol% and 65 vol% at a HDPE/wax ratio of 40/60 with 5 wt% stearic acid as surfactant. As expected, the higher solid load yields a viscosity raise as well. The viscoelastic behavior demonstrated in Figure 13b is more meaningful. The increase of the Ti6Al4V content causes a pronounced increase, especially in the storage modulus, which crosses at moderate shear stresses of the loss modules. This gives clear evidence of the existence of a yield point, which is favorable for FFF printing.

### 3.4. Extrusion-Based Shaping

#### 3.4.1. Fused Filament Fabrication (FFF)

As known from previous work, the filament extrusions of PE-based feedstocks deliver non-elastic filaments that cannot be winded. Consequently, approximately 50 cm long straight filaments have been extruded on a metal cooling track. These filament rods were fed into the printer’s filament conveying system from above. Table 2 covers the best filament extrusion parameters for the different LDPE/HDPE/wax feedstocks. The variation in the individual binder composition caused some adjustments:

- Increasing HDPE amounts led to higher extrusions temperatures;
- Huge LDPE amounts required a smaller extrusion nozzle diameter due to pronounced die swelling;
- Feedstock without LDPE could not be extruded due to enhanced brittleness;
- All feedstocks contained only SA (5 wt%) as surfactant.

**Figure 14:**
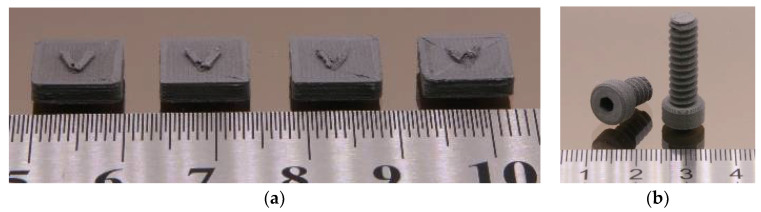
(**a**) FFF-printed cuboids with increasing HDPE content (from left to right: 0%, 15%, 25%, 35%, remaining to 50%: LDPE, wax: 50%, solid load 60 vol%); (**b**) printed screws (feedstock composition: LDPE: 50%, wax: 50%, solid load 60 vol%). In all cases, 5 wt% SA as a surfactant was used.

Table 3 presents an overview of the applied FFF printing parameters delivering the best printing results. The printing process seems to be insensitive to the variation of the feedstock composition, only the system with the highest HDPE amount needs an elevation of the printing temperature. Figure 14a shows (from left to right) the printed cuboids with increasing HDPE contents. The latter ones reduce the achievable quality of the printed structures; the printed body is released from the spring steel during printing, showing pronounced warpage. The best composition consisting of HDPE/LDPE/wax 0/50/50 with 5 wt% SA was used to print more complex parts, such as screws (Figure 14b). Even the screw thread possessed an acceptable quality. The screw was printed in an upright position. FFF printing of feedstocks with higher solid loads than 60 vol% was not possible.

#### 3.4.2. Fused Feedstock Deposition (FFD)

The FFD method enables larger flexibility in the printed feedstock compositions than FFF (Table 4). On the one hand, LDPE-free feedstocks were printable, on the other hand, a solid load of 65 vol% Ti6Al4V was usable. Due to the larger forces in FFD during printing, equivalent to larger shear stresses, the pseudoplastic feedstock flow behavior could be exploited and, hence, the printing temperature could reduce.

**Figure 15:**
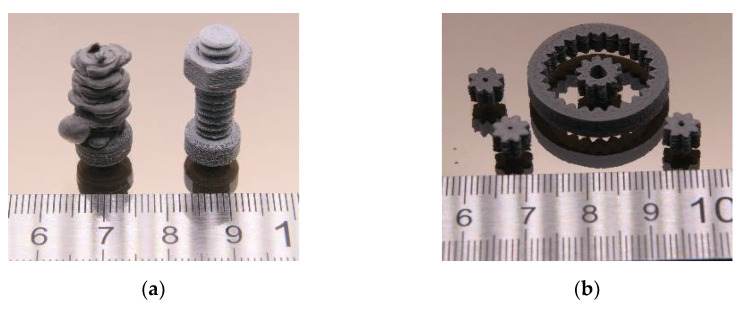
(**a**) FFD printed screw without (**left**) and with additional printed part (**right**) cooling (feedstock composition: HDPE: 40%, wax: 60%, solid load 60 vol%); (**b**) printed planetary gearbox (feedstock composition: HDPE: 25%, LDPE: 25%, wax: 50%, solid load 60 vol%). In all cases, 5 wt% SA as a surfactant was used.

To reduce the warpage, the platform temperature was slightly increased, and all other parameters were quite similar to FFF. The LDPE-free feedstocks were printed at elevated temperatures around 180–190 °C depending on the solid load. Figure 15a shows the influence of the presence of an additional device cooling fan close to the printed part. If additional cooling was not installed, the printing of one layer caused smearing of the previously printed ones (Figure 15a, left). The cooling of the previous printed layers led to solidification, retaining the printed structure (Figure 15a, right), but very fine details, such as teeth in the gearbox (Figure 15b) could not be realized. Up until now, FFD had not been used for the 3D printing of Ti-alloys containing feedstocks.

#### 3.4.3. Metal Injection Molding

**Figure 16:**
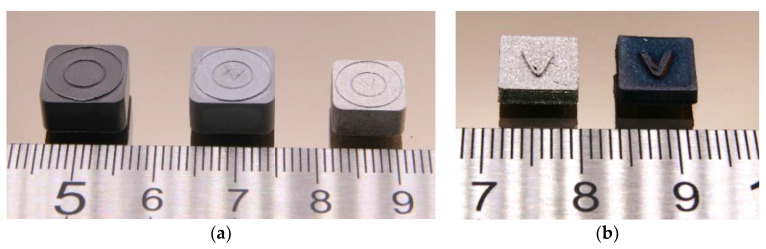
Initial solid load: 60 vol%: Ti6Al4V: (**a**) injection-molded cuboids after molding, liquid debinding, and after sintering (from left to right) demonstrating the part shrinkage while processing. (**b**) Printed cuboids after sintering and hot isostatic pressing.

Metal injection molding of Ti-alloys is an established shaping technique. A comprehensive overview of Ti-alloy MIM with a focus on Ti6Al4V can be found in [52]. With respect to biomedical applications, a comparison between selective laser melting and MIM for the shaping of a complex Ti-alloy was described in [53]. An early work described the MIM of Ti6Al4V by applying a naphthalene/EVA/SA binder. The authors achieved moldable solid loadings up to 65 vol% [54]. Injection molding was conducted with a feedstock composition of HDPE/LDPE/wax of 40/0/60, a solid load of 60 vol%, and 5 wt% stearic acid as surfactant. This feedstock composition was selected for a better comparison of the MIM parts with the FFF and FFD printed ones. The temperature of the injection unit was set to 175 °C, the applied injection pressure was between 150 and 200 MPa with a complete mold filling as the decision criterion. These samples (Figure 16a) were used for comparison with the 3D-printed parts (Figure 16b).

### 3.5. Debinding and Sintering

Debinding, i.e., the removal of the organic feedstock components, was performed as a two-step process. First, liquid debinding in n-hexane was followed by a thermal debinding with its pronounced gas evolution at elevated temperatures. Table 5 lists the relative mass loss during liquid debinding referring to the total wax and SA content for different binder compositions as filaments (FFF) and after shaping (FFD, MIM). For better statistics, 3 filament samples and 10 specimens each were investigated. LDPE-containing feedstocks showed the highest weight loss better than 90%. Increasing HDPE content lowers the dissolution significantly, which was observed by Zhao et al. in the case of n-heptane as a solvent [51]. The binder removal rate described in [51] was lower (<80%), which may be attributed to the bigger n-heptane molecule and different LDPE, HDPE, and wax molecular weights, and/or polymer branching. The authors found a higher wax/SA removal with increasing LDPE content. Due to its branched molecular arrangement, LDPE possesses a lower density and an enhanced free volume allowing easier swelling in contact with small solvent molecules and better wax removal in case of a longer solvent exposition. The absence of LDPE significantly reduces the dissolution of wax/SA below 90%; the relatively large standard deviation can be a hint of an inhomogeneous feedstock composition and phase separation. The results for the injection-molded samples being better may be due to the additional compounding in the screw and the high-pressure material injection. The use of a water-soluble binder system consisting of PEG/PMMA/SA with a Ti6Al4V load of 69.5 vol% enabled a binder removal of almost 100% (sample thickness: 1.5 mm) at 60 °C within 5 h [55]. This rapid debinding process can be attributed to the enhanced attractive interaction between PEG and water at elevated temperatures [55].

Finally, all samples were thermally ‘debinded’ (heating rate: 1.5 K/min, max. temperature 550 °C), followed by a sintering step (heating rate 5 K/min, max. temperature 1350 °C), and finished by cooling down to ambient conditions (rate 15 K/min). Selected samples ran through hot isostatic pressing according to ASTM F3001-14 [56] (heating and cooling rate 15 K/min, max. temperature 920 °C for 2 h, max. pressure 100 MPa, argon atmosphere) to improve the sample density. Figure 16a shows cuboids after injection molding (left), liquid debinding (middle), and sintering (right). Figure 16a presents a sintered printed cuboid (left) with the additional HIP process step (right). The dark blue–violet colored surface appearance of the HIPed sample is obvious, which is a hint of oxygen contamination and the presence of Ti_2_O_3_.

### 3.6. Characterization of Sintered Parts

#### 3.6.1. Part Density and Shrinkage

**Figure 17:**
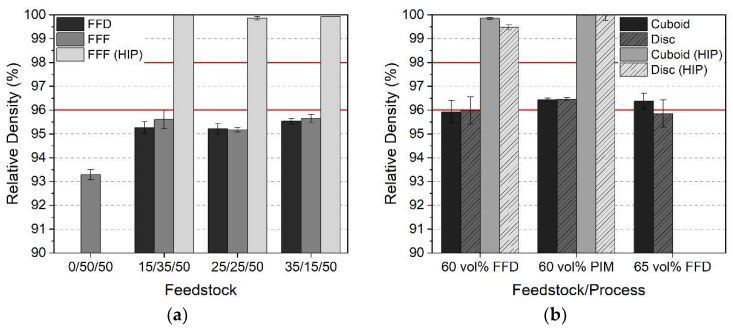
Relative density values after sintering and additional hot isostatic pressing (HIP): (**a**) cuboids made from feedstocks consisting of HDPE/LDPE/wax and a solid load of 60 vol%; (**b**) cuboids and disks made from feedstocks consisting of HDPE/LDPE/wax 40/0/60, variation of shaping method, and a solid load. Red lines according to values of ASTM F2884-17.

The properties of the sintered parts depend on the initial feedstock composition (binder and a solid load), the sintering conditions, and to some extent on the device geometry. Figure 17a shows the achieved density values for different HDPE/LDPE/wax ratios. In all cases, FFF and FFD printing delivered almost identical density values at a solid load of 60 vol%. Only the additional HIP-processed FFF printed samples allowed density values better than 98% (here, 99.3%), which are mandatory values for the usage as medical implants according to ASTM F2885-17 (without HIP: 96%, with HIP: 98%, red lines in Figure 17a) [30].

The variation of the HDPE/LDPE ratio did not show any significant impact on the density. Only the omission of HDPE caused a significant density drop down to 93.3%, which can be attributed to the crack formation during solvent debinding due to pronounced LDPE swelling. Even the presence of small amounts of HDPE suppresses this defect formation. A comparison of the influence of the sample geometry, solid load, and shaping method (FFD and PIM) is presented in Figure 17b. The densities of the cuboids and the discs are almost identical (60 vol% solid load) with values around 96%, but only PIM as a high-pressure molding technique surpasses the ASTM 2885-17 96% limit (red lines in Figure 17b). Additional HIP enabled the best density values—more than 99% of the theoretical density. Surprisingly, a further increase of the solid load to 65 vol% yielded only in the case of the cuboid to a minor density increase or of the disc to a density decrease. Perhaps the printing strategy of the disc-printing favored the void formation, which could not be compensated by the huge infill of 105% due to the large feedstock viscosity and the occurrence of a yield point (Figure 13). This could also be an explanation for the higher mass loss during debinding of the disc (Table 5). Singh et al. [36] reported the best sintered density at 95.6% (solid load: 59 vol%, d_50_: 30 µm), which is in good agreement with the results presented here.

**Figure 18:**
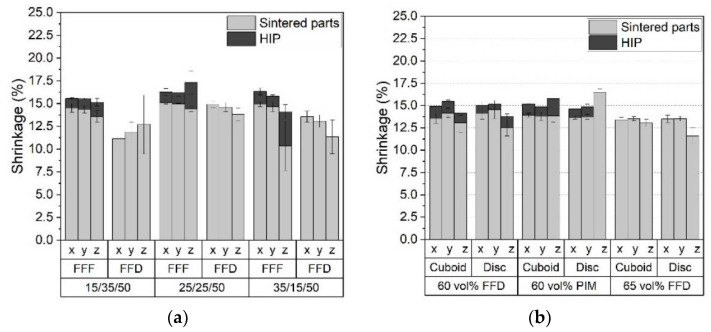
Shrinkage after sintering and additional hot isostatic pressing (HIP): (**a**) cuboid: feedstock consisting of HDPE/LDPE/wax and a solid load of 60 vol%; (**b**) feedstock consisting of HDPE/LDPE/wax 40/0/60, variation of shaping method, and a solid load.

The density values delivered only average information about the shrinkage during sintering. With respect to structural accuracy, a more precise reproduction could be obtained by the comparison of the geometric x,y,z-shrinkage data (Figure 18). With the exception of the FFF-printed cuboid at low HDPE content, a pronounced anisotropic shrinkage can be observed (Figure 18a). In the case of FFF-printing, the in-plane shrinkage (x,y) was almost equal; in the z-direction, mostly a bigger shrinkage can be seen. This difference can be attributed to the non-uniform control system (printhead x,y: rubber belt, platform z: drive screw). In the case of the FFD technique with fixed printheads, the platform was completely moved by belts (x,y) and a drive screw (z). In contrast to FFF, there was a non-uniform deviation in x,y, and z, which may also have its origin in the drive control system. At small HDPE contents, the different impacts of the printing method were obvious. In all cases, the shrinkages of specimens printed by FFD were smaller than by the FFF-printed ones. An additional HIP step increased the shrinkage toward larger values. The effect was in the z-direction (more prominent than in x,y). This can be attributed to closing the pores between the printed layers in a better way than the ones within the x,y printing plane. Figure 18b, for the feedstock HDPE/LDPE/wax of 40/0/60, shows the shrinkages for both types of specimens, printed by FFD or PIM, and the related data for a solid load of 65 vol% in combination with FFD. In the first case, there was no remarkable difference between FFD and PIM, only in the case of the disc could a better value for the z-direction (in the case of PIM) be observed. This may be related to the gate system in the micro-injection molding machine, which injected the molten feedstock parallel to the z-axis followed by an applied holding pressure during the cool down. This caused a pronounced packing of the material, preventing any voids and enabling good sintering behavior. As in the previously shown density (Figure 17b), a further solid load increase to 65 vol% did not lead to any further improvement. In the case of FFD, and at both solids loads (60 vol% and 65 vol%), the shrinkage in the z-direction was for the disc significantly smaller than in the x,y-direction, which may be attributed to the disc-printing strategy generating pores, which could not be closed by sintering. This would also explain the relatively low-density values (Figure 17b).

#### 3.6.2. Microstructure and Mechanical Properties

**Figure 19:**
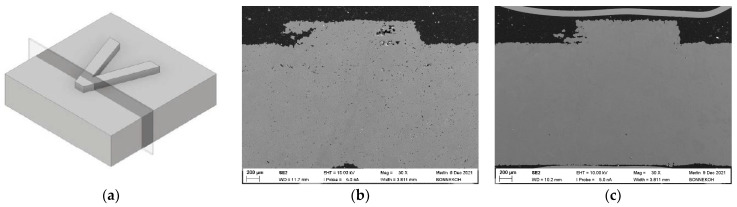
SEM fracture images of the sintered parts (40/0/60 HDPE/LDPE/wax feedstock (solid load: 60 vol%, 5 wt% SA, FFD; (**a**) intersection plane; (**b**) sintered; (**c**) sintered and HIPed.

As discussed earlier, the achieved density values depend on the feedstock compositions and processing steps. Figure 19 shows the impact of the additional HIP-processing step on the microstructure by applying the identical HDPE/LDPE/wax 40/0/60 feedstock. The sintered cuboid achieved a density of 95.7% and the sintered and HIPed—99.8% of theory. Figure 19a shows the used intersection plane and Figure 19b,c show the SEM images of the sintered and sintered/HIPed part. In the latter case, almost all voids were removed.

**Figure 20:**
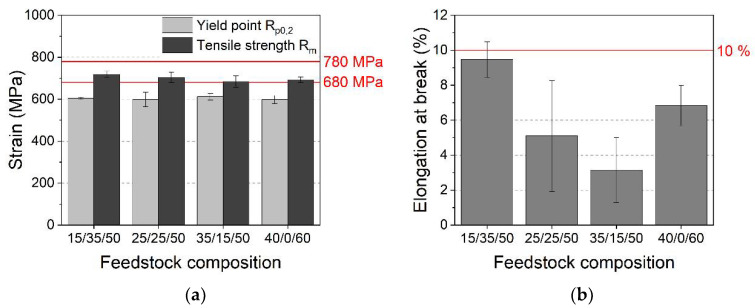
Mechanical properties of the sintered specimen as a function of the initial feedstock composition (solid load: 60 vol%). The red lines represent the requirements of ASTM F2885-17. (**a**) Stress; (**b**) elongation at break.

**Figure 21:**
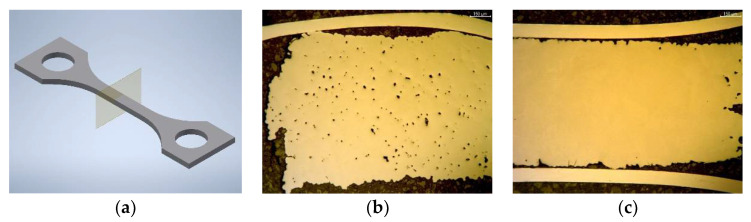
Light microscopic images of fractured surfaces after sanding (solid load: 60%); (**a**) intersection plane; (**b**) feedstock composition HDPE/LDPE/wax: 35/15/50, sintered; (**c**) feedstock composition HDPE/LDPE/wax: 40/0/60, sintered and HIPed.

With respect to the application as a medical implant, an ultimate tensile strength (UTS) of at least 780 MPa, yield strength of 680 MPa, and an elongation at the break of at least 10% for sintered samples were necessary [30]. Figure 20a lists the measured yield strengths and UTS for different initial HDPE/LDPE/wax mixtures. Unfortunately, none of the investigated systems fulfilled the requirements. All mixtures show comparable data; the feedstock with the smallest HDPE content delivered the best values (being approximately 10% below the required thresholds). This system is also very close to the desired elongation at break value (individual specimens achieved the 10% limit, Figure 20b). A clear correlation between feedstock composition and mechanical values could not be found. Figure 21a shows the intersection plane of the specimen, Figure 21b shows a sample made from HDPE/LDPE/wax 35/15/50 (sintered, initial solid load 60 vol%), and Figure 21c shows a sample (sintered and HIPed) made from HDPE/LDPE/wax 40/0/60 (initial solid load 60 vol%). The measured densities were 96% and 99.3% respectively. The 35/15/50 specimen showed a huge number of pores, explaining the poor density value. In the case of the 40/0/60 specimen, the cross-section was almost free from any void. For comparison, Singh et al., for a sample with an initial 30 µm course of Ti6Al4V powder, reported a sintered density of 94.2%, a UTS of around 875 Mpa, and an elongation of 17% [36]. Unfortunately, a direct comparison of the results is difficult because the authors did not supply the complete particle size distribution as well as the specific surface area. Both features affected the final mechanical properties as a result of the compounding and printing properties [36].

#### 3.6.3. Elemental Analysis and Microstructure

**Figure 22:**
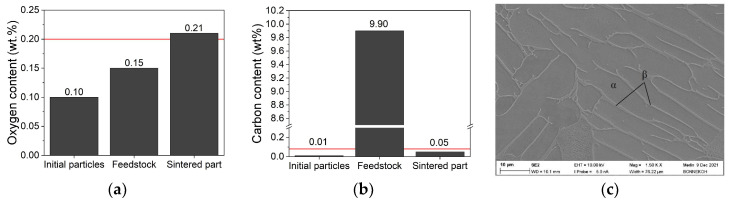
Elemental analysis along processing (initial feedstock composition of LDPE/wax of 50/50 with 60 vol% solid content). The red line represents the maximum allowed values according to ASTM F2885-17 and DIN EN ISO 5832-3:2020-11. (**a**) Carbon content; (**b**) oxygen content; (**c**) SEM image of the sintered and HIPed microstructure (initial composition: HDPE/LDPE/wax 40/0/60; solid load 60 vol%, FFD printing, density 99.8%).

With respect to applications, especially medical devices, the allowed maximum carbon content was set to 0.08 wt% and the maximum oxygen content was fixed to 0.2 wt% [30,57]. In Figure 22, the content of carbon (a) and oxygen (b) was measured along the process chain starting with the initial Ti6Al4V filler, the feedstock system, and the final sintered part. The red lines represent the allowed values according to ASTM F2885-17. In the case of carbon only, the feedstock delivered an extraordinarily large value due to the large organic moiety. The carbon content of the initial particles, as well as of the sintered part, remained below the threshold. In the case of oxygen, a continuous content increase (to a value very low above the allowed threshold) can be observed. This can be attributed to the processing under ambient conditions prior to the debinding step. Scott-Weil reported a carbon content of 210 ppm for the raw powder, which remained almost identical after proceeding with the whole MIM process chain [54]. Singh et al. reported a final oxygen content after sintering of 0.2 wt% or 0.3 wt%, starting with the related initial values of 0.08 wt% and 0.16 wt%, respectively [36]. Figure 22c shows an image of the Ti6Al4V microstructure after etching with ammonium hydrogen fluoride. As expected, a combined α/β lamellar texture can be seen. This agrees with the requirements defined in [30,57].

## 4. Conclusions and Outlook

In a comprehensive approach, the process chain for the additive manufacturing of Ti6Al4V parts via material extrusion methods, such as FFF, and (for the first time) FFD, was developed. The extensive feedstock development and rheological characterization according to the ratio variation of the main binder components, HDPE/LDPE and wax, allowed for tailoring of the feedstock flow properties. The latter are of pronounced relevance for the 3D-printing process (FFF and FFD), because these methods feature low shear rates and small deposition forces, in contradiction to injection molding. Finally, different feedstock systems were developed, which could be used for the fabrication of dense Ti6Al4V parts, almost fulfilling the requirements for the fabrication of medical implants. As in metal injection molding, a general feedstock recipe for different printed part geometries cannot be given because different structural features require different feedstock flow properties. Therefore, small composition adjustments have to be undertaken as needed. Following the measured density and shrinkage data, the solid load should not exceed 60 vol%. Further research will focus on process optimization to improve the mechanical properties according to ASTM F2885-17.

## Figures and Tables

**Table 1:** Particle properties.

Ti6Al4V	Density (g/cm³)	d_10_ (µm)	d_50_ (µm)	d_90_ (µm)	SSA (m²/g)
Measured	4.4	22.1	29.9	39.7	0.15
Vendors certificate	n.a.	21.3	31.5	42.9	n.a.

**Table 2:** Extrusion parameters for different investigated feedstocks (solid load: 60 vol%).

Feedstock Composition: HDPE/LDPE/Wax (%)	Nozzle Diameter (mm)	Extrusion Temperature (°C)
0/50/50	2.7	135
15/35/50	2.7	135
25/25/50	2.7	150
35/15/50	2.8	160
50/0/50	n.a.	n.a.

**Table 3:** FFF-printing parameters for different investigated HDPE/LDPE/wax feedstocks (solid load: 60 vol%, 5 wt% SA as surfactant).

Printing Parameter	0/50/50	15/35/50	25/25/50	35/15/50
Temperature (°C)	170	170	170	180
Platform temperature (°C)	60	60	60	60
Speed (mm/s)	5–10	5–10	5–10	5–10
Speed first layer (mm/s)	5	5	5	5
Nozzle diameter (mm)	0.3	0.3	0.3	0.3
Infill (%)	105	105	105	105

**Table 4:** FFD printing parameter for different investigated HDPE/LDPE/wax feedstocks (solid load: 60 vol%, 5 wt% SA as surfactant).

Printing Parameter	15/35/50	25/25/50	35/15/50	40/0/60	40/0/60 ^1^
Printing temperature (°C) Pellet preheating temperature (°C)	160 120	160 120	170 120	180 120	190 120
Platform temperature (°C)	70	70	70	65	70
Speed (mm/s)	10	10	7.5	5	5
Speed first layer (mm/s)	2.5	2.5	2.5	3	3
Nozzle diameter (mm)	0.4	0.4	0.4	0.4	0.4
Infill (%)	105	105	105	105	105

^1^ Solid load 65 vol%.

**Table 5:** Relative mass loss of wax and stearic acid in n-hexane after 24 h and at 40 °C of different investigated HDPE/LDPE/wax feedstocks (solid load: 60 vol%) and specimen geometries.

Composition	Filament (%)	Disk (%)	Cuboid (%)	Tensile Specimen (%)
0/50/50	97.5 ± 0.3	96.4 ± 1.1	96.6 ± 1.2	-
15/35/50	96.8 ± 0.4	95.6 ± 0.8	95.4 ± 0.4	94.4 ± 0.9
25/25/50	95.6 ± 0.4	95.4 ± 0.9	96.1 ± 0.9	93.1 ± 1.7
35/15/50	94.3 ± 1.7	94.5 ± 0.7	94.3 ± 1.3	92.6 ± 1.0
40/0/60	-	78.1 ± 2.6	82.8 ± 5.2	71.7 ± 2.2
40/0/60 ^1^	-	84.0 ± 2.3	90.5 ± 5.6	90.1 ± 6.8
40/0/60 ^2^	-	88.6 ± 0.9	81.8 ± 0.7	-

^1^ Solid load 65 vol%; ^2^ for comparison: metal injection-molded parts.

## Data Availability

Not applicable.
